# Stromal ColXα1 expression correlates with tumor-infiltrating lymphocytes and predicts adjuvant therapy outcome in ER-positive/HER2-positive breast cancer

**DOI:** 10.1186/s12885-019-6134-y

**Published:** 2019-11-01

**Authors:** Chaohui Lisa Zhao, Kamaljeet Singh, Alexander S. Brodsky, Shaolei Lu, Theresa A. Graves, Mary Anne Fenton, Dongfang Yang, Ashlee Sturtevant, Murray B. Resnick, Yihong Wang

**Affiliations:** 1 Department of Pathology and Laboratory Medicine, Rhode Island Hospital and Lifespan Medical Center, Warren Alpert Medical School of Brown University, 593 Eddy St, APC 12, Providence, RI 02903 USA; 2 Department of Pathology and Laboratory Medicine, Women and Infant Hospital, Warren Alpert Medical School of Brown University, Providence, RI 02903 USA; 3 Department of Surgery, Rhode Island Hospital and Lifespan Medical Center, Warren Alpert Medical School of Brown University, Providence, USA; 4 Department of Medicine, Rhode Island Hospital and Lifespan Medical Center, Warren Alpert Medical School of Brown University, Providence, USA

**Keywords:** Collagen, Tumor infiltrating lymphocytes, Tumor microenvironment, Breast cancer, Adjuvant chemotherapy

## Abstract

**Background:**

The breast cancer microenvironment contributes to tumor progression and response to chemotherapy. Previously, we reported that increased stromal Type X collagen α1 (ColXα1) and low TILs correlated with poor pathologic response to neoadjuvant therapy in estrogen receptor and HER2-positive (ER+/HER2+) breast cancer. Here, we investigate the relationship of ColXα1 and long-term outcome of ER+/HER2+ breast cancer patients in an adjuvant setting.

**Methods:**

A total of 164 cases with at least 5-year follow-up were included. Immunohistochemistry for ColXα1 was performed on whole tumor sections. Associations between ColXα1expression, clinical pathological features, and outcomes were analyzed.

**Results:**

ColXα1 expression was directly proportional to the amount of tumor associated stroma (*p* = 0.024) and inversely proportional to TILs. Increased ColXα1 was significantly associated with shorter disease free survival and overall survival by univariate analysis. In multivariate analysis, OS was lower in ColXα1 expressing (HR = 2.1; 95% CI = 1.2–3.9) tumors of older patients (> = 58 years) (HR = 5.3; 95% CI = 1.7–17) with higher stage (HR = 2.6; 95% CI = 1.3–5.2). Similarly, DFS was lower in ColXα1 expressing (HR = 1.8; 95% CI = 1.6–5.7) tumors of older patients (HR = 3.2; 95% CI = 1.3–7.8) with higher stage (HR = 2.7; 95% CI = 1.6–5.7) and low TILs. In low PR+ tumors, higher ColXα1 expression was associated with poorer prognosis.

**Conclusion:**

ColXα1 expression is associated with poor disease free survival and overall survival in ER+/HER2+ breast cancer. This study provides further support for the prognostic utility of ColXα1 as a breast cancer associated stromal factor that predicts response to chemotherapy.

**Electronic supplementary material:**

The online version of this article (10.1186/s12885-019-6134-y) contains supplementary material, which is available to authorized users.

## Background

Breast cancer is the second leading cause of cancer-related death in women. HER2 targeting therapies, such as trastuzumab and pertuzumab, prolong survival in breast cancer patients [1, 2]. HER2-positive breast cancers constitute a heterogeneous disease at the molecular level. The repertoire of somatic genetic alterations in these tumors varies according to estrogen receptor (ER) status and correlates with “intrinsic” subtype [3–6]. Early studies focusing on breast cancer molecular subtypes showed that Luminal-B subtype has poorer outcome than luminal-A subtype. In fact, the overall survival in untreated luminal-B subtype is similar to basal-like and HER2-positive tumors, which are widely recognized as high risk [3]. The treatment outcome for patients with ER+/HER2+ cancer, sometimes referred to as Luminal HER2 type, is variable [7–9]. Therefore, additional biomarkers are needed for risk stratification and treatment response prediction. We previously reported that increased stromal Type X collagen α1 (ColXα1) expression in ER+/HER2+ tumors is associated with poor response to neoadjuvant therapy. Our findings provided initial evidence that a specific stromal collagen subtype in the breast tumor microenvironment predicts therapy response [10].

Tumor associated stroma is composed of a matrix of fibronectin, matrix metalloproteinases, collagens and other connective tissue proteins which undergo constant remodeling [11]. Tumor-stromal interactions play a key role in tumorigenesis and metastasis influencing the prognosis of several human malignancies [12–14]. Some studies have reported that tumor microenvironment components like tumor infiltrating lymphocytes (TILs) and tumor-associated stroma predict response to therapy and breast cancer progression [15]. In this study we aimed to evaluate the prognostic and predictive value of ColXα1 expression in the tumor stroma of ER+/HER2+ breast tumors in the adjuvant setting.

## Methods

### Patients and tissue samples

The study was approved by the ethics committees of Lifespan Medical Center (467617–9) and Women Infants Hospital (797108–3). The need for consent was waived by the IRB.

A retrospective search was performed in the cancer registry database for breast cancer patients who received adjuvant therapy at Lifespan Medical Center and Women and Infant Hospital in Rhode Island between 2007 and 2013. We identified 164 ER+/HER2+ cases who received adjuvant chemotherapy and HER2-targeted therapy. All original tumor slides were reviewed and histological features were recorded. Immunohistochemistry for ER, PR, and HER2 expression were classified according to the CAP/ASCO guidelines [16, 17]. ER and PR positive cases were further classified as low-positive (1–10%) and positive (> 10%).

### Immunohistochemistry and ColXα1 expression scoring

**Fig. 1:**
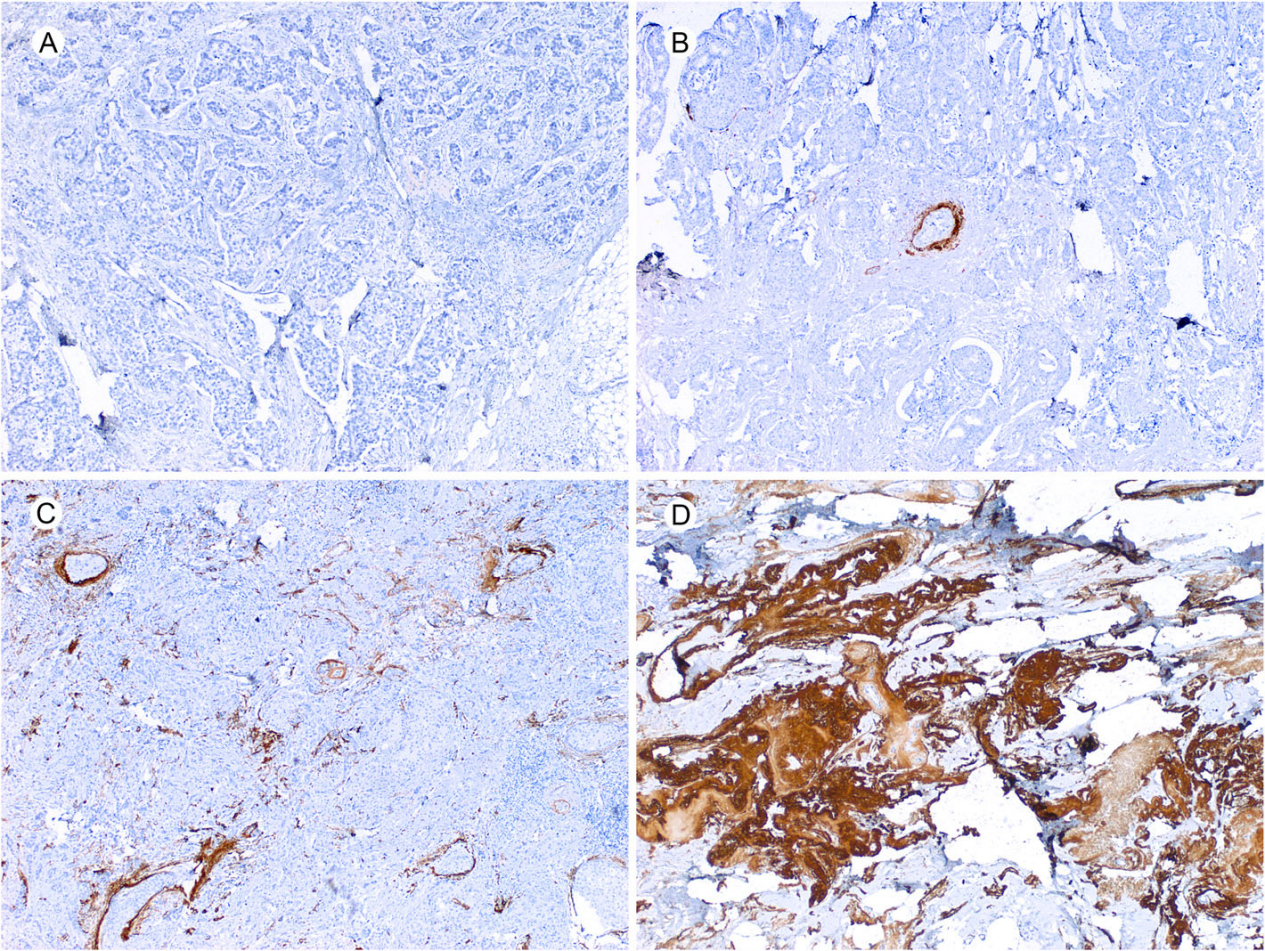
Photomicrographs of stromal ColXα1 immunohistochemical staining (40X). **a** No expression (score 0). **b** Weak expression (score 1). **c** Moderate expression (score 2). **d** Strong expression (score 3) (× 200)

For all cases, 4-μm-thick tissue sections were cut from formalin-fixed paraffin-embedded tumor tissue and subjected to immunohistochemical staining according to the manufacturers’ protocol as previous described [10]. Anti-ColXα1 (1:50, eBioscience/Affymetrix, Clone X53), ER (1:50, DAKO, clone 1D5), PR (1:400, DAKO, clone 1A6), HER2 (DAKO HercepTestTM), and monoclonal mouse anti-human Ki-67 (clone MIB1, Ready-to-use, Dako) were used for immunohistochemistry. ColXα1 was scored as previously described [10]. Briefly, no staining was scored as 0; weak staining as 1+; < 10% of stroma tissue with intense staining present as 2+; > 10% of stroma tissue with intense staining as 3+ (Fig. 1). ColXα1 immunohistochemical stain was further grouped into low (scores 0–1) and high expression (scores 2–3) categories. Ki-67 was scored as percentage of tumor cells with nuclear staining by two pathologists (YW and KS) independently on fresh cut slides. The Ki-67 score was calculated for each patient by averaging the two pathologists readings.

### Tumor-associated stroma and TILs analysis

We morphologically evaluated the amount of intratumoral stroma and TILs on tumor samples. The stromal evaluation protocol has been previously published [10]. Briefly, the amount of tumor-associated stroma was scored as 0 to 2: 0 for absent or minimal stroma (< 10%), 1 for mild to moderate amount of stroma (10–40%) and 2 for abundant stroma (> 40%). The TILs were evaluated based on criteria published by Denkert et al. [15]. Briefly, iTILs are defined as lymphocytes in direct contact with the tumor cells, whereas sTILs are defined as lymphocytes in the surrounding stroma with the percent of the tumor or stromal volume comprised of infiltrating lymphocytes. The results were evaluated in increments of 10 (0–1% was scored as 0, with all other estimates rounded up to the next highest decile [for e.g. 11–20% was scored as 20]). sTILs and iTILs were added to calculate TILs. The trends were similar for each lymphocyte fraction (data not shown). We chose to analyze sTILs as they are considered as the most consistent metric (as recommended by the International TILs Working Group) [18]. While analyzing survival, the sTILs data was divided into three groups. Group 1: Rare TILs in the stroma (0–10%). Group 2: low density TILs (11–59%) and Group 3: high density TILs (> = 60%).

### Gene expression and pathway analysis

TCGA RNA-seq data for breast invasive carcinoma was downloaded from the Firehose Broad GDAC [19]. TCGA clinical data was downloaded from the TCGA data archive in September 2015 (http://cancergenome.nih.gov/).

### Statistical analysis

Fisher Exact test and Chi-square test were used when appropriate. T-test or analysis of variance (ANOVA) was used to compare continuous variables. For time-to-event measures, the Kaplan–Meier method was used to estimate the empirical survival and log-rank estimates were used. Relationships between variables were assessed using Pearson and Spearman correlation analysis as noted. Multivariate analysis was performed using the Cox proportional hazards model. Statistical analysis was performed utilizing *SPSS v25 (SPSS, Chicago, IL, USA).* A *P*-value < 0.05 was considered statistically significant. All *P*-values reported are two-sided.

## Results

### Patient characteristics and clinical pathologic features

A total of 164 ER+/HER2+ breast cancer patients who received adjuvant therapy and with at least a five year follow up were included in the study. All data generated or analyzed during this study were de-identified and included in this published article (Additional file 1: Table S1). The clinical and pathologic data of the patients are summarized in Table 1. Mean age was 60 years (median = 58; range = 25–99). There were 142 (87%) ductal, 9 (5%) lobular and 13 (8%) mixed ductal and lobular carcinomas. Out of 164 cases, 120 (73%) underwent lymph node sampling including 86 (72%) axillary dissections and 34 (28%) sentinel lymph node biopsies. There were 66 (40%) grade 2, 95 (58%) grade 3 and 3 (2%) grade 1 tumors. Lymphovascular invasion (LVI) was identified in 95/130 cases (73%) and lymph node metastasis were present in 56/120 (47%) cases. As expected for the ER+/HER2+ group, there were 87 (53%) stage II, 52 (31%) stage I and 22 (13%) stage III cases. Out of 164 cases, 86 (52.4%) had only rare TILs, 57 (34.8%) had low density TILs and 21 (12.8%) tumors showed high density TILs. The Ki-67 IHC was performed on 144 cases, of which 133 (95%) tumors had a Ki-67 index > = 20% (median = 40, range = 20–90) consistent with a luminal B intrinsic subtype. Seven (5%) tumors with Ki-67 index estimated < 20% were positive for ColXα1. The mean Ki-67 index for grade 2 tumors (34.8; 95% CI = 29.8–39.9) was significantly lower than grade 3 tumors (44.9; 95% CI = 40.5–49.3).

**Table 1:** Clinicopathological characteristics of cohort. Fisher’s exact test and Chi-square test (as appropriate) were used to generate *P*-values

Characteristic	No.	ColXα1 Positive N (%)	P-value
No. of patients	164	62.8%	
Age (year)	164		
< 65	99	57 (57.5%)	0.10
≥ 65	65	46 (71%)	
Age (year)			
< 58		61%	0.75
≥ 58		64%	
Laterality	164		
Left	94	54 (57.3%)	0.52
Right	70	30 (42.7%)	
Foci	164		
Solitary	146	61.6%	0.45
Multifocal	18	72.2%	
LVI	130		
Pos	97	62 (63.9%)	0.005
Neg	33	21 (63.6%)	
Lymph node status	120		0.21
N0	64	30 (46.9%)	
N1	42	26 (61.9%)	
N2	8	4 (50.0%)	
N3	6	5 (83.3%)	
Clinical stage	164		
I	52	61.5%	0.34
II	87	60.9%	
III	22	77.3%	
IV	3	33.3%	
ColXα1	164		
0	21		–
1	40		
2	47		
3	56		
Stroma Content	164		
0	30	15 (50%)	0.024
1	95	57 (60%)	
2	39	31 (79.5%)	
TILs	164		
1 (Rare)	22	15 (68.2%)	< 0.001
2 (Low density)	113	82 (72.6%)	
3 (High density)	29	6 (20.7%)	

### Correlation of ColXa1 with other factors and TILs

Overall ColXα1 positivity was present in 143/164 (87%) cases. ColXα1 expression was directly proportional to the amount of stroma in the tumor (*p* = 0.024) and correlated with the presence of LVI (*p* = 0.008). There was a significant inverse relationship between TILs and ColXα1 expression by immunohistochemistry (r = − 0.47; *p* <  0.001, Table 2). A significantly higher proportion of tumors with rare and low density TILs were ColXα1 positive when compared to the high density TILs group (*P* <  0.001, Table 1). ColXα1 expression was present in 15/22 (68.2%) cases with rare TILs. Similarly, 82/113 (72.6%) of tumors with low density TILs were ColXα1 positive. Conversely, only 6/29 (20.7%) high density TILs cases had ColXα1 expression.

**Table 2:** Spearman and Pearson Correlations of ColXα1 with Tumor Immune Environment

Characteristic	Pearson with ColXα1	P-value
RIH dataset	ColXα1 IHC	
TILs	−0.47	< 0.001
Age	0.099	0.21
TCGA (ER/HER2 only, *N* = 102) Spearman with ColXα1 RNA-seq		
Naïve B Cells	−0.29	0.003
CD 8 T Cells	−0.36	< 0.001
Monocytes	−0.28	0.005
Lymphocytes	−0.41	< 0.001
Macrophages	0.40	< 0.001
TIL Percentage	−0.01	0.32

### Correlation of ColXa1 and TILs and tumor associated immune cells

In the TCGA cohort, a similar trend of inverse relationship between the tumor immune microenvironment and ColXα1 expression by RNA-seq was noted (Table 2). There was an inverse relationship between ColXα1 and lymphocytes (r = − 0.33; *p* = 0.001), Naïve B cells (r = − 0.22; *p* = 0.02), CD8 T cells (r = − 0.24; p = 0.02) and monocytes (r = − 0.26; *p* = 0.008). A positive correlation was present between ColXα1 RNA-seq and macrophages (r = 0.35; *p* < 0.001). The TCGA cohort, like our group, showed an inverse relationship between TILs and ColXα1, however the *p*-value did not reach statistical significance (r = − 0.15; *p* = 0.1).

### ColXα1, TILs and survival

**Fig. 2:**
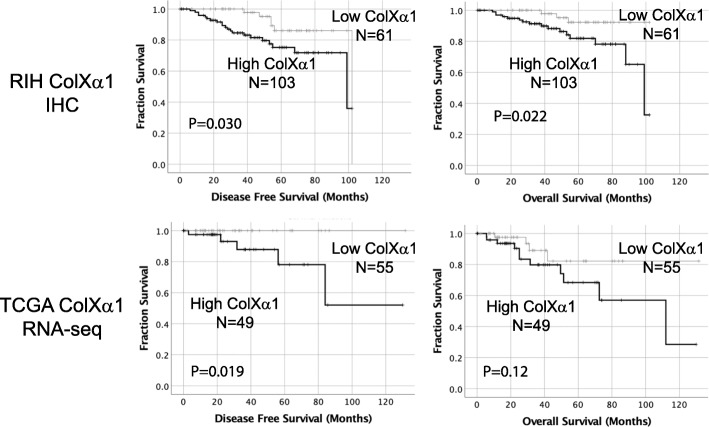
Survival analysis and ColXα1 expression by IHC in current study (top). The DFS and OS were significantly different between high ColXα1 (103 patients) and low ColXα1 (61 patients) ER+/HER+ tumors. Survival analysis in the TCGA ER+/HER2+ tumor dataset (bottom).. Patients stratified into higher (49 patients) and lower (55 patients) than the median of COL10A1 mRNA expression showed significantly different DFS and OS

**Fig. 3:**
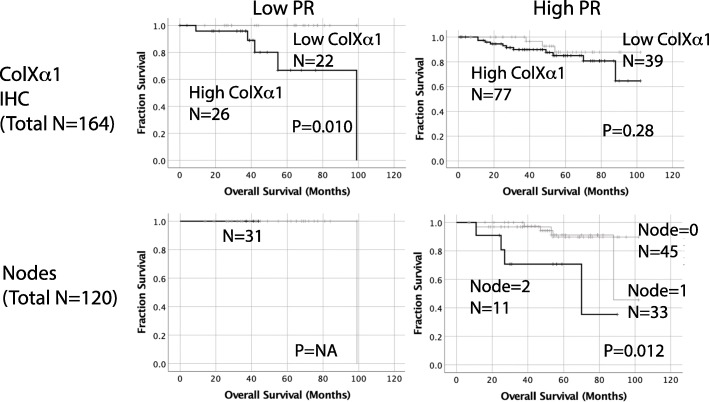
Interaction and Impact of PR status, ColXα1 expression and number of metastatic nodes on survival. ColXα1 expression levels stratify only low PR+ tumors into prognostic groups (top left). Presence of lymph node metastasis was not a risk factor for patients with low PR levels but was a risk factor for patients with high PR levels. Nodes were grouped as follows: Node score = 0, 0 nodes, Node score = 1, 1–3 nodes, Node score = 2, > 3 nodes

A statistically significant difference in breast cancer disease free survival (DFS) (HR = 1.8; 95% CI, 1.6 to 5.7) and overall survival (OS) was found between high and low ColXα1 tumors (HR = 2.1; 95% CI, 1.2 to 3.9) by cox proportional hazards analysis (Table 3). Similar survival trends were noted in the TCGA cohort (Fig. 2). In a multivariate cox proportional hazards analysis, OS was lower in ColXα1 expressing (HR = 2.1, 95% CI, 1.2 to 3.9) older patients (HR = 5.3, 95% CI, 1.7 to 17) with higher stage (HR = 2.6, 95% CI, 1.3 to 5.2) and low TILs (HR = 0.96; 95% CI, 0.9–1.0). Similarly, DFS was lower in ColXα1 expressing (HR = 1.8, 95% CI, 1.6 to 5.7) older patients (HR = 3.2, 95% CI, 1.3 to 7.8) with higher stage (HR = 2.7, 95% CI, 1.6 to 5.7) and low TILs (HR = 0.98; 95% CI, 0.94–1.0) (Table 4). We noted a relationship between PR status, ColXα1 and OS in our cohort. In low PR group of 48 (29%) tumors, OS was significantly lower in ColXα1 high tumors. ColXα1 expression was associated with poor outcome in low PR tumors but not high PR tumors by stratification Kaplan-Meier analysis (Fig. 3).

**Table 3:** Cox Proportional Hazards Univariate Analysis (*N* = 158 unless otherwise noted). 95% confidence levels are indicated in the parentheses for the Hazard Ratios (HR)

Characteristic	HR OS	P OS	HR PFS	P PFS
Clinical Stage	2.0 (1.1–3.4)	0.02	2.1 (1.4–3.3)	< 0.001
Grade	1	1.0	1.1 (0.5–2.3)	0.77
Age	1.08 (1.05–1.1)	< 0.001	1.05 (1.0–1.1)	< 0.001
Lymph Node Status (*N* = 120)	2.5 (1.4–4.5)	0.002	2.7 (1.6–4.4)	< 0.001
PR status	1.1 (0.4–3.1)	0.83	1.1 (0.5–2.7)	0.76
Stroma	1.1 (0.6–2.3)	0.73	1.1 (0.6–2.0)	0.72
TILs	0.96 (0.92–1.0)	0.027	0.97 (0.9–1.0)	0.025
ColXα1	2.3 (1.3–4.1)	0.006	1.8 (1.2–2.8)	0.008

**Table 4:** Multivariate analysis of variables with *p* < 0.05 and significant HR as univariate for overall and progression free survival (N = 158). 95% confidence levels are indicated in the parentheses for the Hazard Ratios (HR)

Factor	HR OS	P OS	HR PFS	P PFS
Age (> 58)	5.3 (1.7–17.0)	0.005	3.2 (1.3–7.8)	0.01
ColXα1	2.1 (1.2–3.9)	0.01	1.8 (1.6–5.7)	0.01
Clinical Stage	2.6 (1.3–5.2)	0.008	2.7 (1.6–4.7)	< 0.001

## Discussion

The HER2+ breast cancers are a heterogeneous group with variable tumor aggressiveness and response to therapy [20, 21]. HER2+ tumors can be further classified according to the hormone receptor status. Luminal-B subtype is defined as ER+ tumor with increased tumor cell proliferation which is usually assessed by KI-67 [22]. The ER+/HER2+ tumors are also classified as luminal-B subtypes, and sometimes referred to as Luminal HER2 subtype [6]. In our cohort of Luminal B HER2 subtype, most tumors (95%) had high KI-67 expression (> = 20%). Although some studies report better outcome in Luminal HER2 breast cancer when compared to ER-negative HER2-positive breast cancer [20–23], others have found no significant difference in long term survival [24–26]. Metastatic sites and recurrence patterns of HER2+ tumors are ER dependent. Bone is the most common site of distant metastasis for Luminal HER2 subtype, which is similar to Luminal A cancers, whereas ER-negative HER2-positive cancers have the higher rates of locoregional recurrence and these tend to initially metastasize to visceral organs such as lung [23, 26, 27]. In ER+/HER2+ breast cancer, HER2 overexpression predicts poor response to hormone therapy [28, 29]. Cross talk between HER2 and ER results in persistent activation of the hormone receptor downstream signaling, even in the presence of hormone treatment [30].

The tumor microenvironment consists of non-cellular elements such as collagens, glycosaminoglycans, proteoglycans and hyaluronic acid. Collagens are major components of the extracellular matrix of breast tumors. Collagen expression patterns have been linked with aggressive tumor behavior and drug resistance [31]. Together with hyaluronic acid, collagen accumulation is also associated with high tumoral interstitial pressure, vascular collapse and drug resistance [32, 33]. Myriad collagen genes are critical in tissue development and physiological function, as evidenced by the range of collagenopathies [34]. Nevertheless, the mechanistic role of collagen subtypes in cancer progression has been largely overlooked, although they frequently emerge as components of tumor stromal expression signatures [35, 36]. A variety of collagen subtypes are highly expressed in breast tumors contributing to its dense structure. The alignment of collagen fibers has been proposed to indicate progression in breast tumors [37, 38]. ColXα1and its hexagonal network that plays a role in tissue stiffness has been associated with chemoresistance and breast cancer progression [39]. Collagen type X mRNA expression is up-regulated in a variety of human malignancies when compared to normal tissue, including breast tumors [40]. In colorectal cancer, Huang and colleagues demonstrated that ColXα1 expression was significantly higher in the tumor stroma compared with normal tissues. ColXα1enhanced proliferation, migration, invasion of colon cancer cells and knockdown of ColXα1 inhibited tumorigenesis in vivo [41]. Similar to the findings in the colorectal tissue, we reported that ColXα1 was not expressed in normal breast tissue [10]. Interestingly ColXα1 is present in a periductal location surrounding ductal carcinoma in situ, and within the tumor associated stroma in select invasive breast carcinomas, particularly in ER+/HER2+ cancers [42].

We had earlier identified that presence of ColXα1, determined by gene expression analysis and immunohistochemistry, predicted neoadjuvant therapy response in ER+/HER2+ tumors. In the current study, we demonstrated that ColXα1 expression predicts long term outcome in ER+/HER2+ tumors in the adjuvant setting as well. In our cohort most of the patients were treated before the results of the Z-11 trial were published and axillary dissection was performed in a significant number of cases. Consistent with other studies [3, 7–9], ER+/HER+ tumors exhibited aggressive features; three quarters of the cases had LVI, and half of the cases had lymph node metastasis.

We found an inverse relationship between TILs and ColXα1 expression, and that low TILs and ColXα1 both contribute to the poor treatment response and prognosis. We did not found differences in TIL or ColXα1 expression with age. The relationship of ColXa1 expression and imaging findings, like mammographic density, can be investigated in future studies.

In addition, ER and PR positivity is defined by > 1% of nuclear staining of the tumor cells. In practice, a large amount of information is lost when one labels a tumor as a ER or PR-positive, because a tumor in which 10% of cells exhibit weak ER or PR staining is biologically different from one that demonstrates strong intensity staining in about 90% of cells. Although the vast majority of hormone receptor positive tumors show strong immunoreactivity, approximately 20% of tumors exhibit variable ER/PR expression. Raghav et al. [43] demonstrated no significant impact on survival and benefit of endocrine therapy in low ER/PR+ cases, which was similar to triple negative tumors. In the NSABP B-14 clinical trial Baehner and coworkers reported greater benefit from tamoxifen in patients with higher ER expression [44]. We analyzed our cases based on ER/PR expression and found that in the low PR+ group of 48 (29%) tumors, OS was significantly lower in ColXα1 high tumors. ColXα1 expression was associated with poor outcome in low PR+ tumors but not in high PR tumors. Like PR, other tumor stromal factors and tumor microenvironment variables, such as ColXα1 have a potential to predict the treatment response and outcome.

## Conclusion

Our findings indicate that extent of ColXα1 expression in the ER+/HER2+ breast tumor stroma is prognostic. Increased ColXα1 expression is associated with shorter OS and DFS in both neoadjuvant as well as adjuvant settings. Further molecular or proteomic studies are needed to refine the definition of ColXα1 positive/overexpressing tumors. The relationship of TILs and ColXα1is intriguing, which needs further investigation, including correlation with imaging studies.

## Additional file

Additional file 1: **Table S1.** Raw data for all cases in this study All data generated or analyzed during this study were included and de-identified (XLSX 26 kb)

## Data Availability

All clinical pathological relevant information is summarized in Table 1 and raw data of all case was provided in Additional file 1: Table S1.

## Abbreviations

ColXα1: Type X collagen α1
DFS: Disease free survival
ER: Estrogen receptor
HER2: Human epidermal growth factor receptor 2
IHC: Immunohistochemistry
OS: Overall survival
TILs: Tumor infiltrating lymphocytes
